# Annual Thermal Stress Increases a Soft Coral’s Susceptibility to Bleaching

**DOI:** 10.1038/s41598-019-44566-9

**Published:** 2019-05-30

**Authors:** Marc Slattery, M. Sabrina Pankey, Michael P. Lesser

**Affiliations:** 1 University of Mississippi, Department of BioMolecular Sciences, Oxford, MS 38677 USA; 2 University of New Hampshire, Department of Molecular, Cellular, and Biomedical Sciences, Durham, NH 03824 USA; 3 University of New Hampshire, School of Marine Science and Ocean Engineering, Durham, NH 03824 USA

**Keywords:** Climate-change ecology, Marine biology

## Abstract

Like scleractinian corals, soft corals contain photosymbionts (Family Symbiodiniaceae) that provide energy for the host. Recent thermal events have resulted in soft coral bleaching in four of five years on Guam, where they dominated back-reef communities. Soft coral bleaching was examined in *Sinularia maxima*, *S*. *polydactyla*, and their hybrid *S*. *maxima* x *polydactyla*. Results from annual field surveys indicated that *S*. *maxima* and the hybrid were more susceptible to bleaching than *S*. *polydactyla*, and this was related to differences in their Symbiodiniaceae communities in 2016 and 2017. The photosymbionts of *S*. *polydactyla* were apparently more stress tolerant and maintained higher photosynthetic potential through three years of bleaching, in contrast to the other species that exhibited a decline in photosynthetic potential after the first year of bleaching. Nonetheless, by the 2017 bleaching event all soft coral populations exhibited significant bleaching-mediated declines and loss of photosynthetic efficiency suggesting a declining resiliency to annual thermal stress events. While *S*. *polydactyla* initially looked to succeed the other species as the dominant space occupying soft coral on Guam back-reefs, cumulative bleaching events ultimately turned this “winner” into a “loser”, suggesting the trajectory for coral reefs is towards continued loss of structure and function.

## Introduction

The consequences of climate change on coral reefs in the coming decades are expected to be significant^1,2^. Among the projected impacts, extreme storm events^3^ can cause serious damage to coral reefs^4^, and ocean acidification is expected to affect a variety of coral physiological processes^5^. However, the most significant climate change effect will be the increase in global sea surface temperatures^6^ that has resulted in an increased incidence of “coral bleaching” at a number of sites worldwide^7–9^. Thermal stress, often combined with high irradiance, causes photo-oxidative stress and the loss of symbiotic dinoflagellates (Family Symbiodiniaceae) from host coral tissues resulting in the bleached appearance of affected colonies^10^. The symbiotic dinoflagellates have recently been reorganized from the current cladal designation (*i*.*e*., A-I) to different genera in the Family Symbiodiniaceae^11^, and where appropriate the new taxonomy will be used here. Susceptibility to thermal stress varies temporally and spatially^12,13^, as well as among coral species^14,15^, with photosymbiont lineage considered an important contributing factor^16^. Depending on both host characteristics^17,18^ and symbiont taxon^19^, exposure to elevated seawater temperatures can lead to thermal acclimation or mortality of the holobiont.

The loss of photosymbionts represents a significant energetic cost to the host coral that can manifest as reduced colony fitness^20^. However, bleached corals can survive and recover on energetic reserves by switching to heterotrophy for short periods of time^17,21^, until the symbiont community is re-established^22^. Alternatively, corals and their symbionts have the ability to acclimatize to chronic thermal stress providing potential future resilience to bleaching^20,23^. Grottoli *et al*.^24^ addressed the role of acclimatization over two consecutive bleaching years, with three species of scleractinian corals, in a hybrid laboratory-field experiment. They demonstrated that the recovery of colonies following a single bleaching event, resulting in acclimatization of corals to bleaching in the subsequent year, is species-, and symbiont-, specific. Therefore, predicting the “winners” and “losers^25^” from the cumulative effects of thermal stress will be problematic since susceptibility to future bleaching events can result in wholescale coral mortality and/or changes in community structure and function^26^. Currently, multiple bleaching events in any one location have been shown to result in communities dominated by non-scleractinian structural groups such as algae, sponges, and soft corals^27^. But soft corals, for example, are not necessarily an alternate stable state; instead they are crucial members of the coral reef community that may become increasingly important with the loss of scleractinian structure and function^28^.

Like their hard-coral relatives, soft corals also have a symbiotic relationship with members of the Family Symbiodiniaceae that is an important source of energy that augments soft coral heterotrophic feeding^29^. Unlike scleractinian corals, the trophic mode of soft-corals is largely mixotrophic^30^. While there is apparently less symbiont diversity within soft corals relative to hard corals^31^, this symbiont diversity, either low or high, can determine whether a particular species trophic mode is primarily autotrophic or heterotrophic^32^. Similar to scleractinian corals, soft-corals can acquire their symbionts through vertical or horizontal transmission^33^, and they appear to be more stable through time and space than scleractinian-Symbiodiniaceae associations^34^. Moreover, the loss of these symbionts has significant impacts on biochemical characteristics of adult soft corals and their eggs^35^, with consequences for reproductive potential^36^.

*Sinularia* spp. is one of the most common genera of soft corals in the Indo-Pacific and it provides habitat and food, while contributing to the consolidation of the reef^28^. On the leeward reefs of Guam, *S*. *maxima*, *S*. *polydactyla*, and their hybrid *S*. *maxima* x *polydactyla*^37^ have been the most abundant soft corals for at least two decades^38^. The demographics of these soft coral populations have varied through time due to differential susceptibilities to predation, competition, sedimentation, and disease^37,39^. The last reported bleaching event on Guam occurred in November 1994, when Slattery & Paul^40^ documented mild bleaching of the soft coral *S*. *maxima*. That study provided evidence for differential susceptibility to bleaching within *S*. *maxima* populations, and among species as *S*. *polydactyla* did not bleach in the same event^41^. As thermal stress and subsequent bleaching events have become increasingly common on Guam^42^, we present the population dynamics and bleaching susceptibility of three soft coral populations relative to associated changes in their symbiont communities.

## Results

### Annual temperature profile, soft coral cover, and bleaching prevalence

**Figure 1:**
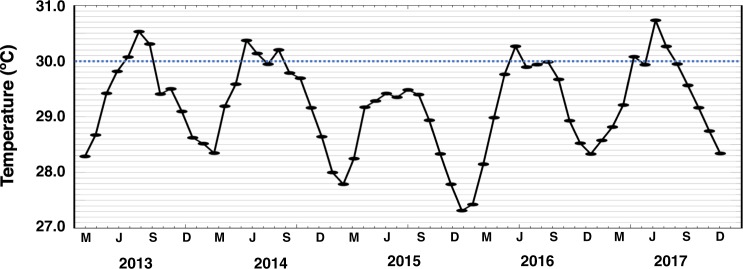
Sea surface temperatures for Guam. Plotted are the mean monthly SSTs (°C) from March 2013 to December 2017 using NOAA Coral Reef Watch products. The night-time Advanced Very High Resolution Radiometer (AVHRR) SST data provided near real-time 5-km thermal profiles for the study site. The dashed line equates to the regional bleaching threshold value, defined by NOAA CRW as 1 °C above the mean temperature of the warmest month in the regional seasonal cycle.

**Figure 2:**
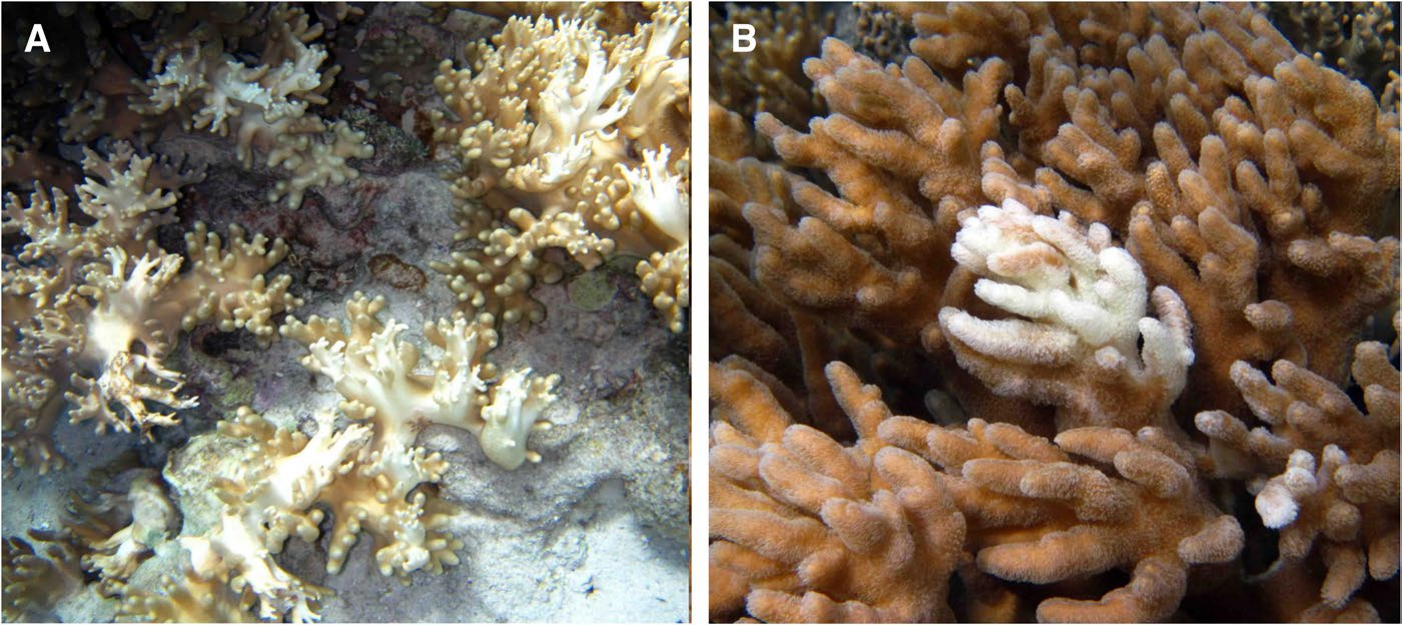
Representative bleaching in *Sinularia* spp. Presented are: (**A**) “complete” bleaching in *S*. *maxima*, and (**B**) “partial” bleaching in *S*. *polydactyla*. The bleached surface area of the *S*. *maxima* colonies (photo area = 1 m^2^) are >70%, while the bleached surface area of *S*. *polydactyla* colonies (photo area = 10 cm^2^) are <1%. Completely bleached soft corals almost always resulted in colony mortality, while partially bleached soft corals rapidly recovered. Note: the hybrid soft coral *S*. *maxima* x *polydactyla* (not shown) exhibits a bleaching pattern similar to *S*. *maxima*.

**Figure 3:**
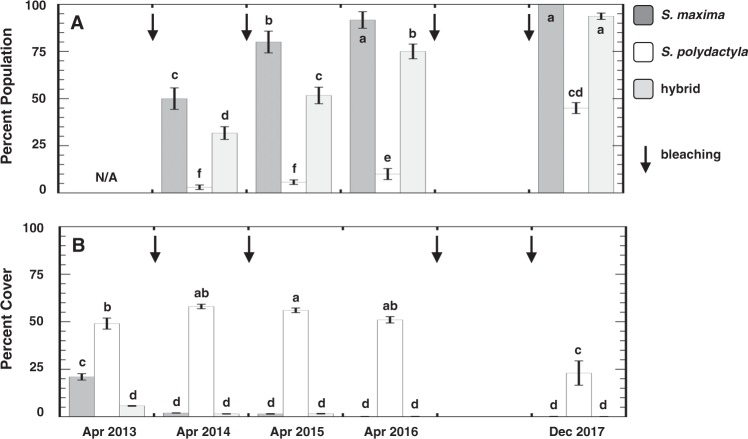
Soft coral populations in Guam. Histograms represent the mean ± 1SE (**A**) percent of each soft coral population that was bleached between April 2013 and December 2017, and (**B**) percent cover of each soft coral population at PBH Guam during that period. Different letters above histograms indicate significant differences by ANOVA. Arrows (↓) correspond to approximate periods when sea surface temperature exceeded the bleaching threshold. See Fig. 1 for approximate dates of the bleaching events each year (note: bleaching threshold line), and Table S1 for results of post hoc analyses on significant two-way ANOVAs. N/A indicates no bleached colonies in April 2013.

The plotted monthly average SSTs for Guam indicate that four of five recent years have exceeded the regional bleaching threshold (Fig. 1). Associated with these rising SSTs were observations of bleaching within the three populations of soft corals at PBH (Fig. 2); and onset of bleaching was correlated with the 4 to 8 Degree Heating Weeks thresholds (Fig. S1, and reports from eormarianas.org). It is possible these bleaching events were exacerbated by irradiance (Fig. S2) as the DHWs occasionally overlapped with doldrum periods of 3 to 7 days duration (Fig. S3). Specifically, in April 2014 (6+ months after the 2013 thermal anomaly) 50% and 32% of the *S*. *maxima* and hybrid populations, respectively, were bleached (Fig. 3a). In subsequent years, these populations exhibited a significant increase in the percent of individuals that bleached until December 2017 when almost all of these soft corals were affected (two-way ANOVA_year x species_: F_4,2_ = 22.3811; P < 0.0001). In contrast, *S*. *polydactyla* exhibited significantly less susceptibility to these bleaching temperatures with only ~2–10% bleached through April 2016 (Fig. 3a). However, there was a significant increase in *S*. *polydactyla* bleaching susceptibility after the 2016 thermal anomaly; 45% of the population was bleached in December 2017 (Tables S1 and S2). There was a significant decline in the populations of *S*. *maxima* and the hybrid immediately following the bleaching event during the summer of 2013 (two-way ANOVA_year x species_: F_4,2_ = 55.2158; P < 0.0001), with percent cover dropping from 21% and 5%, respectively, to ~1% (Fig. 3b). In contrast, the population of *S*. *polydactyla* initially increased by ~5–10%, before it ultimately dropped to half the 2013 population level in December 2017 (Table S1 and S2).

### Soft coral health status

**Figure 4:**
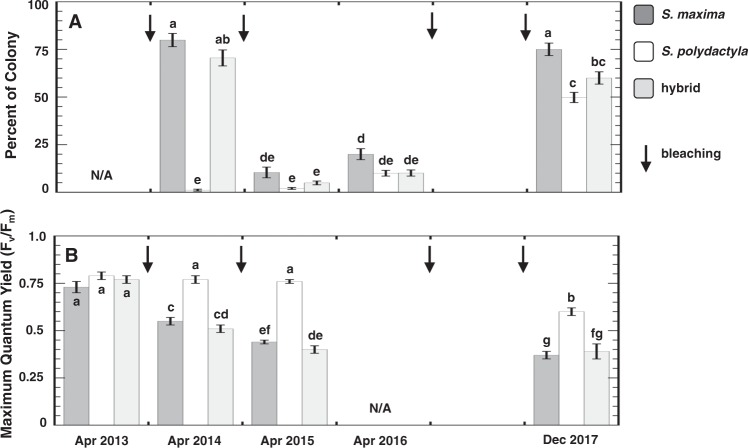
Soft coral populations in Guam. Histograms represent the mean ± 1SE (**A**) percent of each soft coral colony [n = 15] that was bleached between April 2013 and December 2017, and (**B**) average quantum yield of each soft coral colony [n = 15] at PBH Guam during that period. Different letters above histograms indicate significant differences by ANOVA. Arrows (↓) correspond to approximate periods when sea surface temperature exceeded the bleaching threshold. See Fig. 1 for approximate dates of the bleaching events each year (note: bleaching threshold line), and Table S2 for results of post hoc analyses on significant two-way ANOVAs. N/A indicates no bleached colonies in April 2013, and no PAM data in April 2016.

The percent bleaching over the surface of the soft coral colonies varied through time (Fig. 4a). Following the 2013 thermal anomaly, 70–80% of the surface of *S*. *maxima* and hybrid colonies were bleached (Fig. 2a), whereas <1% of the surface of *S*. *polydactyla* exhibited bleaching (Fig. 2b). However, between the 2014 and 2016 thermal anomalies, there was a significant change in percent bleaching for all three soft corals ranging from 3–20% (two-way ANOVA_year x species_: F_3,2_ = 53.9766; P < 0.0001). By December 2017 the percent bleaching for colonies of all three soft corals increased to ~50–75%. Significantly, the bleaching for *S*. *polydactyla* in 2017 represented at least a six-fold increase in susceptibility relative to prior years (Fig. 4a; Table S3). With the increase in soft coral bleaching through time, there was a significant decline in photosynthetic efficiency, measured as the maximum quantum yields of PSII fluorescence (Fig. 4b; two-way ANOVA_year x species_: F_3,2_ = 58.0471; P < 0.0001). Prior to the thermal anomaly during the summer of 2013, the average quantum yield of all three soft corals was ~0.75, and *S*. *polydactyla* maintained that photosynthetic efficiency through April 2015 despite two intervening bleaching events (Fig. 1). Interestingly, there was a significant decline in the average quantum yield of *S*. *polydactyla* by December 2017 (Table S3). In contrast, the photosynthetic efficiencies of *S*. *maxima* and the hybrid declined by a third after the first thermal anomaly alone in 2013, and remained relatively consistent through time afterward (Fig. 4b).

### Family symbiodiniaceae taxonomy

Each ITS2 library yielded 33711 ± 15656 (mean ± 1 SD) merged reads following initial processing and quality control steps. ITS2 variant analysis using SymTyper assigned the vast majority of reads to clade C, now all in the genus *Cladocopium*. Only 11 reads from all assessed (1,136,264 reads) were assigned to other clades (ten to clade B and one to clade G). Of this total, 2.1% (23,777 reads) did not meet the cut-off to match Hidden Markov Model profiles for any clade type. The symbiont communities appeared to be dominated by a small number of common phylotypes, although these phylotypes differed between *Sinularia* species. The most common phylotype CL_172 (*Cladocopium* C71a) previously reported from the scleractinian coral *Orbicella*^43^ constitutes ~69% of the communities in *S*. *maxima* and the hybrid, but only 2% of the *S*. *polydactyla* symbiont communities. Several other phylotypes recovered from these samples also fall in this clade, although they represent much smaller proportions of the symbiont community and were not differentially abundant among species, with the exception of phylotype CL_200 (*Cladocopium* sp.) that was enriched in *S*. *maxima* and the hybrid. Other common variants CL_170 (*Cladocopium* C1017) and CL_174 (*Cladocopium thermophilum*) together account for 51% of *S*. *polydactyla* reads, but only 6% of *S*. *maxima* and hybrid reads (Fig. S4; Table S4). Given the limited phylogenetic resolution provided by ITS2 for the Family Symbiodiniaceae^44^ and the high genetic diversity of the genus *Cladocopium*, phylogenetic assignment can be tenuous for many newly recovered phylotypes (Fig. S5) in the absence of a more comprehensive phylogenetic study (*sensu* 11). Overall, the majority of the Symbiodiniaceae enriched *in S*. *polydactyla* are all in the genus *Cladocopium*, and include *C*. *goreaui* and *C*. *thermophilum*. In contrast, the majority of the Symbiodiniaceae enriched in *S*. *maxima* and the hybrid are more phylogenetically restricted (*i*.*e*., less diverse), placing them within, or proximate to *Cladocopium* C71a. Raw MiniSeq reads from ITS2 amplicon libraries are available under NCBI BioProject accession PRJNA504909.

**Figure 5:**
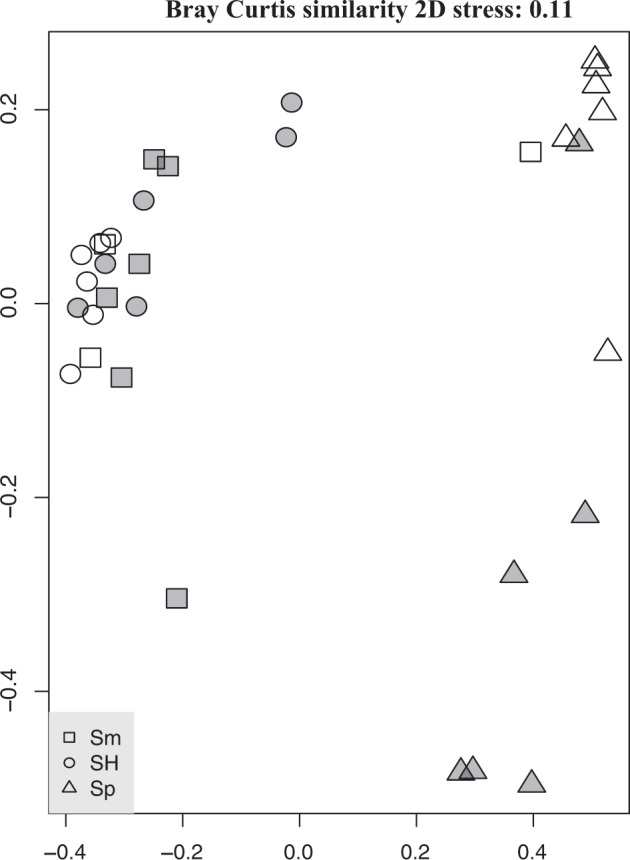
Soft coral photosymbiont communities. Nonmetric MDS plot of the photosymbiont composition across the soft corals *Sinularia maxima* (Sm), *S*. *polydactyla* (Sp), and their hybrid (SH) in each of two bleaching years, 2016 (white symbols) and 2017 (grey symbols), based on normalized read abundances of each Symbiodiniaceae variant recovered from MiSeq (Table S4). Data indicate that symbiont communities are more similar in *S*. *maxima* and the hybrid, than in *S*. *polydactyla* (Table S5), and that this soft coral’s photosymbiont community change coincides with reduced resilience.

For the members of the Symbiodiniaceae there are significant effects for both host species and collection year. Additionally, their interaction was significant and accounted for over 50% of the community variation observed (Table S1). For both years, more Symbiodiniaceae variation is shared between *S*. *maxima* and the hybrid than with *S*. *polydactyla* (Fig. 5; Table S5). Collection year had the greatest effect on *S*. *polydactyla*, driving significant changes in beta diversity (Tables S3, S6; Fig. 5), while Symbiodiniaceae beta diversity was significantly higher in *S*. *polydactyla* (one-way ANOVA_species_: F_2_ = 5.2; p = 0.007) than in *S*. *maxima* or the hybrid. Overall, alpha diversities did not differ significantly across species or year (Table S2). Symbiodiniaceae community variation among the 2017 samples was not significantly impacted by experimental treatment (i.e., caged = predator exclusion; Table S7).

## Discussion

Seawater temperatures conducive to coral bleaching have become increasingly common on the reefs of Guam over the past five years, exceeding predictions for thermal anomaly cycles in the western Pacific^45^. While regional surveys have revealed the severity of these bleaching events^42^, recovery of bleaching impacted populations in the region have largely been overlooked (but see^40^). In addition, differential susceptibilities to bleaching, within and between species, have only been addressed in a qualitative, observational manner^41^. Importantly, this study demonstrates significant bleaching impacts to the soft coral community of Piti Bomb Holes (PBH) Guam, with evidence for species-specific responses and changing resilience through time.

The three soft coral populations studied here responded differently to thermal stress. Specifically, *S*. *maxima* and the hybrid exhibited “complete bleaching” relative to *S*. *polydactyla*, that exhibited “partial bleaching”, when the first bleaching event was observed in 2013. Coincident with the increased susceptibility to bleaching in *S*. *maxima* and the hybrid, was a significant decline in photosynthetic efficiency and increased mortality, indicating sensitivity to thermal stress in these two soft coral populations. In contrast, the limited bleaching, higher photosynthetic efficiency, and stable/increased population growth in *S*. *polydactyla* indicated a soft coral population that was resistant to thermal stress when this study began. Taken as a whole, these data support observations of species-specific bleaching differences in corals generally (*e*.*g*.^14,20,24,25,46^), and in these soft corals specifically^40,41^. Moreover, these differences were manifested at the community level; in recent years *S*. *polydactyla* has increased in percent cover within the PBH soft coral community relative to *S*. *maxima* and the hybrid^38^. Although the competitive dominance of *S*. *polydactyla* predates the bleaching events, and was primarily due to increased resistance to a soft coral disease^39^, this soft coral’s thermal tolerance was clearly important in maintaining, and expanding, its population size when sea surface temperatures (SSTs) increased on Guam in recent years. It is worth noting that the soft corals observed throughout this study were likely the most stress-tolerant individuals within their respective populations, as they also survived continuous anthropogenic sedimentation and the presence of a unique soft coral disease^37,39^. The fact that these stress-tolerant soft coral populations ultimately succumbed to temperature-mediated bleaching reinforces the implications of continuing increases in thermal stress to coral reef communities^1,9^.

The increased susceptibility to bleaching in *S*. *polydactyla* during 2017, following four years of resistance to thermal stress, demonstrate that soft coral resilience can change through time. Prior to 2013, Guam reefs had not bleached in about two decades^42^, when *S*. *maxima* bleached and *S*. *polydactyla* did not^40,41^. The lack of a response in *S*. *polydactyla* colonies during that 1994 event might have been due to differences in bleaching severity at the time (*sensu*^47^). Paulay & Benayahu^41^ noted that thermal stress was likely not a major factor in the 1994 bleaching event. Slattery & Paul^40^ indicated that bleaching on Guam may have been due to enhanced solar radiation (*i*.*e*., bleaching occurred at depths <1 m after two weeks of doldrum and cloudless conditions), which significantly enhances oxidative stress and the molecular cascade of events leading to apoptosis and bleaching compared to increased SSTs alone^10^. Since 2013, regional bleaching has been tightly coupled to rising SSTs which has a much more ubiquitous influence on shallow coral reef communities in space, and time^9^. Specifically, the duration of the thermal stress event, and its interaction with other stressors such as solar radiation, can further influence the bleaching response of a specific species^24,48^. For example, experimental bleaching studies of hard corals, greater than one month in duration, demonstrated species-specific differences in recovery rates, relative to PSII repair, indicative of variable resilience in Hawaiian corals^49^. Significantly, the longer recovery times of corals exposed to extended bleaching events increase the likelihood that those individuals will be susceptible to consecutive bleaching events^24,50^. Our sampling periods through 2016 were typically six to eight months after the thermal anomalies and the soft corals were still heavily bleached indicative of slow recovery periods and the likelihood of cumulative thermal stress. Although this might also suggest some seasonal variation in bleaching onset (i.e., April vs. December sampling periods), and potentially coupling with other stressors of soft corals^37–39^. In addition, the photosymbionts of *S*. *polydactyla* did change through time (i.e., 2016 to 2017), which offers support for the role of symbiont-mediated resilience to climate change stressors (e.g.^6,24^).

Strychar *et al*.^46^ documented differential bleaching susceptibilities in three genera of soft corals on the Great Barrier Reef, including an unidentified species of *Sinularia*, and suggested this might be due to thermal acclimation and/or differences in heterotrophic feeding. The trophic relationship between soft corals and their photosymbionts is arguably important^35,36,40^, although likely less so than that of hard corals and their symbionts^51^. This is similar to other non-calcifying taxa in the Anthozoa, such as sea anemones, where photosymbionts often supply less than 50% of the carbon requirements of the host^52^. *Sinularia polydactyla* has larger polyps than *S*. *maxima* and the hybrid (Slattery pers. obs.), so it is possible that some of the differences in resilience may be due to enhanced heterotrophic efficiency (*i*.*e*., increased encounter and capture rates) in *S*. *polydactyla* after bleaching events (*e*.*g*., 17). However, the differences in photosymbiont loss (*i*.*e*., complete vs. partial bleaching) between *S*. *maxima* and the hybrid, and *S*. *polydactyla*, respectively, suggests that thermal adaptation (*e*.*g*., different symbiont species) may have been an important factor in the initial resilience of *S*. *polydactyla* (*e*.*g*.^53^), although it is also possible that the thermal stress, and subsequent bleaching, was less severe. Nonetheless, there is some evidence for thermal acclimatization in *S*. *maxima* and the hybrid as well. Following the 2014 bleaching event, *S*. *maxima* and the hybrid exhibited significant reductions in the percent of colony areal bleaching relative to colonies during the 2013 bleaching event. It is likely that the surviving soft coral populations had acclimatized to thermal stress, and therefore were not as susceptible to bleaching during this period. However, the return of bleaching temperatures in 2016 and 2017 resulted in a further decline in resilience manifested as a reduced percent cover of the PBH soft coral community.

The importance of differences in symbionts from the Family Symbiodiniaceae relative to bleaching resistance and resilience has been well documented for hard corals^20,54,55^, and even some soft corals (*e*.*g*.^56^). The differences in bleaching susceptibility between *S*. *maxima* and the hybrid, and *S*. *polydactyla*, are significantly correlated with their respective dominant symbionts. Specifically, all three soft corals have clade C photosymbionts from the genus *Cladocopium*, as do most soft corals in the Pacific^31^, but the beta diversity is also significantly higher in *S*. *polydactyla* relative to *S*. *maxima* and the hybrid indicative of distinct symbiont communities within these soft corals. Furthermore, the symbionts enriched in *S*. *polydactyla* span a greater phylogenetic range, consistent with greater potential breadth of ecological niches that could promote host resistance and resilience when exposed to thermal stress^57^. It appears likely there are unique photosymbionts in *S*. *polydactyla* that are more heat-tolerant than those in *S*. *maxima* and the hybrid^46,58^, (but see^59^), although it is also possible that host tolerance^25^ host-symbiont interactions^60^, host energy stores measured as lipid concentration^21^, and/or post-bleaching feeding capacity^17^ are responsible for the thermal resistance observed in *S*. *polydactyla*. Overall, these three soft coral populations hosted 71 unique members of the Symbiodiniaceae, and individual soft coral colonies hosted in excess of 30 members of this family. Photosymbiont community diversity has been shown to vary across environmental gradients^61,62^, and within individuals relative to incident irradiance environments^63^. The soft coral habitat of PBH Guam, and the soft coral morphologies, are relatively consistent^37^, so it is unlikely that environmental gradients, or predators, structure the symbiont communities within the PBH soft coral populations. Instead, the differences in soft coral photosymbiont communities are likely a function of the *in hospite* physiological conditions of each soft coral population (*i*.*e*., host specificity: 54), and possibly due to the nutrients each host provides to the symbionts through reverse translocation^64^.

Thermal stress has become increasingly common on coral reefs of the western Pacific requiring acclimatization by regional coral communities^20,23^. Stress-tolerant photosymbionts in the soft coral *S*. *polydactyla* were apparently the primary mechanism by which this population was able to contend with multiple bleaching events (*e*.*g*.^19^), and to successfully gain space while two conspecific populations declined^38^. But these populations eventually succumbed to thermal stress as well; *Sinularia polydactyla* has faced thermal stress in four out of the last five years, as well as continued natural and anthropogenic stressors^37,39^, and this has resulted in reduced resistance and resilience, population density, and physiological health, as well as increased mortality. Grottoli *et al*.^24^ showed that cumulative thermal stress events could turn some coral “winners into losers” and some “losers into winners”, all with implications for community structure and function. Here we present evidence that shallow coral reef soft corals that were “winners” ultimately become “losers” as the upper ceiling for the ability to recover and/or acclimatize to thermal stress is reached, and exceeded. It is clear there is some degree of species-specific resistance and resilience relative to climate change impacts that sets up a winners and losers scenario^25^. But if current climate change scenarios in PBH Guam, and the three dominant soft corals at that site, are indicative of regional trends in community compositions, it’s possible that many of the soft coral communities of the western Pacific will be lost, with critical implications for coral reef structure and function^9,38^.

## Materials and Methods

### Annual oceanographic profiles

The SST and DHW data from Guam during March 2013 to December 2017 were recovered from NOAA Coral Reef Watch thermal stress monitoring products^65^. Specifically, the night-time Advanced Very High Resolution Radiometer (AVHRR) SST data, collected from the NOAA Polar-Orbiting Environmental Satellites (POES), provided near real-time 5-km thermal profiles for the study site^66^. The mean monthly maximum SSTs were plotted against the regional bleaching threshold value^67^; notably, the bleaching events in 2013, 2014, and 2017 were of similar intensity providing insights into soft coral resilience. Average daily windspeed at PBH was plotted against the NOAA-defined doldrums value (3 m^−s^)^65^. Average monthly irradiance values were collected hourly between May 2016 and December 2017 using HOBO Pendant dataloggers (n = 3) on the backreef of PBH (*e*.*g*.^68^).

### Soft coral cover and bleaching prevalence

Soft corals were monitored at Piti Bomb Holes, Guam (PBH: 13°28.10′N, 144°42.00′E), where their landscape ecology has been extensively studied^37–39^. Eight 30 × 30 m permanent grids were established in this shallow (1–3 m depth) back-reef flat, and all colonies within the grids were mapped and tagged to assess individual health status through time^37^. Due to a disease-mediated loss of soft coral cover within these grids^39^, in 2013 we established 10 × 2 m band transects (n = 5) over the remaining soft coral patches within each of the eight original grids. The percent cover of *S*. *maxima*, *S*. *polydactyla*, and *S*. *maxima* x *polydactyla*, and the percent of bleached individuals within each population (*i*.*e*., bleaching prevalence) were recorded annually between 2013 and 2017.

### Soft coral health status

Tagged soft corals (n~100 of each species) at PBH were followed though time and a subset (n = 15 of each species) that survived were included in an assessment for percent of colony bleached annually between 2014 and 2017. A 0.25 m^2^ quadrat was strung to provide 25 equidistant points to estimate percent cover using point intercept methods. The quadrat was held above each colony and to the side of each colony to estimate horizontal planar and vertical planar projected surface area, and to calculate percent areal bleaching for each soft coral colony.

To assess the species-specific responses to bleaching in the three soft coral populations, active fluorescence was measured in the aforementioned tagged colonies using a pulse-amplitude modulated (PAM) fluorometer (Walz Inc.) in 2013, 2014, 2015, and 2017. PAM measurements were recorded along the “fingers” of each soft coral colony (see Fig. 2 for example). Soft coral measurements (n = 3 per colony) were taken from the same distance, probe angle, and instrument settings at dawn, ensuring dark acclimation^69^. Minimum (F_o_) and maximum (F_m_) fluorescence were used to calculate variable fluorescence (F_v_ = F_m_ − F_o_), and subsequently the maximum quantum yield of photosystem II (PSII) fluorescence (F_v_/F_m_) or the number of functional photosystem II reaction centers^69^.

### Family Symbiodiniaceae taxonomy

Symbiodiniaceae diversity, based on ITS2 diversity, was sampled in *S*. *maxima*, *S*. *polydactyla*, and the hybrid in 2016 and 2017. In 2016, replicate colonies of each species representing healthy (n = 3), and bleached *S*. *polydactyla* and the hybrid (n = 3), were collected at PBH. In 2017, replicate healthy colonies of each species (n = 3) were caged to prevent predation effects^40^, while paired healthy colonies of each species (n = 3) served as uncaged controls. Genomic DNA was extracted from the *Sinularia* samples (~150 mg blotted tissue) using the PowerSoil Kit (Qiagen) following the manufacturer’s protocol with the following modifications. Prior to the initial tissue disruption, samples were incubated for 3 hours at 55 °C with 0.5 mg Proteinase K (Qiagen) added to the kit’s 750 ml PowerBead solution. Tissue disruption was enhanced by the addition of 100 μl glass beads (600–800 µm, Sigma).

To amplify the ITS2 fragments, 50–70 ng gDNA were added to PCR reaction tubes containing AmpliTaqGold 360 + GC Enhancer (ThermoFisher) and 0.5 µM of each Fluidigm-tagged primer (CS1_ITS_DinoF: 5′-aca ctg acg aca tgg ttc tac agt gaa ttg caa gaa ctc cgt g, CS2_ITS2rev2: 5′-tac ggt agc aga gac ttg gtc tcc tcc gct tac ttt aat atg ctt)^70,71^. Reactions were heated to 95 °C for 5 min before runs of 30 cycles at 95 °C (30 s), 52 °C (45 s), 72 °C (45 s), and a final extension of 7 min at 72 °C. The ~340 bp amplicons were sequenced on an Illumina MiSeq (2 × 250) at the Center for Genomic Research DNA Services Facility (University of Illinois-Chicago).

### Raw data processing of ITS2 libraries

Demultiplexed raw data were processed as follows: (1) MiSeq read pairs were merged using PEAR (-p 1.0 -m 450 -n 25^56^), (2) merged reads were filtered by quality using prinseq (-min len 250 -max_len 450 -min_qual_score 20 -min_qual_mean 30 -noniupac -log: 57), and (3) predicted chimeric sequences were removed using chimera_filter.pl with a reference database of ITS2 sequences from the package ‘microbiome helper^72^’.

### Characterization of Symbiodiniacea diversity

The Family Symbiodiniaceae sequence diversity was quantified with the aid of scripts from SymTyper (https://github.com/UH-Bioinformatics/symTyper^43,44^), a bioinformatic pipeline developed for characterizing *Symbiodinium* spp. ITS2 sequences. Using Hidden Markov Model profiles for *Symbiodinium* spp. ITS2 sequences, SymTyper assigned sequences to *Symbiodinium* spp. clade level (*e*.*g*., A through I), with clade assignment based on an e-value cutoff of 10^−20^ and contingent upon an e-value at least 10^−5^-fold better than the next best clade hit. To quantify within-clade diversity, sequences were then subsequently clustered at 99% similarity using cd-hit-est^73^.

Downstream analyses used read counts of each Symbiodiniacea cluster assignment. Only clusters with reads represented in over 30% of samples were retained for analysis. Read counts were scaled by smallest library size prior to statistical tests. Scaled counts were square-root transformed for visualization purposes only.

### Phylogenetic analysis

The Clade C (*Cladocopium*) reference ITS2 database used by SymTyper^44^ was clustered to 99% similarity using cd-hit-est^73^ to retain sufficient diversity for species resolution in the diverse C clade^74^. Database clusters and the unique *Sinularia* ITS2 sequence clusters were then aligned with MAFFT^75^. A maximum likelihood phylogeny was inferred using RAxML under the model GTRGAMMA^76^. Bipartition support was inferred using 500 bootstrap replicates.

### Statistical analyses

For the three species of soft coral, the percent cover, bleaching prevalence, and percent colony bleaching data were all arcsin transformed and tested using two-way analysis of variance (ANOVA) with interaction, with species and year as fixed factors. In addition, the maximum quantum yield of PSII (*i*.*e*., F_v_/F_m_) data were log transformed and tested using two-way analysis of variance (ANOVA) with interaction, with species and year as fixed factors.

Alpha and beta diversity of the Symbiodiniaceae were measured using the Shannon-Weaver and Sørensen indices, respectively, as implemented in the vegan R package^77^. Differences in diversity between species were assessed using two-way ANOVA. Symbiodiniaceae community composition across samples was ordinated using Kruskal’s non-metric multidimensional scaling method as implemented by the isoMDS function in the MASS R package^78^. Bray-Curtis distances between samples were calculated prior to nMDS using normalized read counts mapped to each of the unique ITS2 sequences recovered and clustered at 99% similarity with CD-HIT. A permutational multivariate analysis of variance (PERMANOVA) tested the effects of species and collection year on Symbiodiniaceae community structure using the ‘adonis’ function in the vegan R package with 9999 permutations^77^. *Post-hoc* PERMANOVAs were also conducted to ascertain pairwise differences between species. Individual ANOVAs were conducted to determine the effect of species and collection year on relative abundances of each member of the Symbiodiniaceae. Significance values were corrected for multiple testing using the Bonferroni method. PERMANOVA was also employed to test for effects of experimental manipulation (*i*.*e*., caged or uncaged) of Symbiodiniaceae among the 2017 soft coral colony samples.

## Supplementary information

Supplementary Info

## References

[1] Hoegh-Guldberg O, Bruno JF (2010). The impact of climate change on the world’s marine ecosystems. Science.

[2] Pandolfi JM, Connolly SR, Marshall DJ, Cohen AL (2011). Projecting coral reef futures under global warming and ocean acidification. Science.

[3] Emanuel KA (2013). Downscaling CMIP5 climate models shows increased tropical cyclone activity over the 21st century. Proc. Natl. Acad. Sci..

[4] Hughes TP (1994). Catastrophes, phase shifts, and large-scale degradation of a Caribbean coral reef. Science.

[5] Doney SC, Fabry VJ, Feely RA, Kleypas JA (2009). Ocean acidification: the other CO2 problem. Ann. Rev. Mar. Sci..

[6] Baker AC, Glynn PW, Riegl B (2008). Climate change and coral reef bleaching: An ecological assessment of long-term impacts, recovery trends and future outlook. Est. Coastal Shelf Sci..

[7] McClanahan TR (2007). Western Indian Ocean coral communities: bleaching responses and susceptibility to extinction. Mar. Ecol. Prog. Ser..

[8] Heron SF, Maynard JA, van Hooidonk R, Eakin CM (2016). Warming trends and bleaching stress of the world’s coral reefs 1985–2012. Sci. Reports.

[9] Hughes TP (2018). Spatial and temporal patterns of mass bleaching of corals in the Anthropocene. Science.

[10] Lesser, M. P. Coral bleaching: causes and mechanisms. In *Coral reefs: An Ecosystem in Transition* 405–419 (Springer, 2011).

[11] LaJeunesse TC (2018). Systematic revision of Symbiodiniaceae highlights the antiquity and diversity of coral endosymbionts. Current Biology.

[12] Fitt WK, McFarland FK, Warner ME, Chilcoat GC (2000). Seasonal patterns of tissue biomass and densities of symbiotic dinoflagellates in reef corals and relation to coral bleaching. Limnol. Oceanogr..

[13] Glynn PW, Mate JL, Baker AC, Calderon MO (2001). Coral bleaching and mortality in Panama and Ecuador during the 1997–1998 El Niño–Southern Oscillation event: spatial/temporal patterns and comparisons with the 1982–1983 event. Bull. Mar. Sci..

[14] Marshall PA, Baird AH (2000). Bleaching of corals on the Great Barrier Reef: differential susceptibilities among taxa. Coral Reefs.

[15] Stimson J, Sakai K, Sembali H (2002). Interspecific comparison of the symbiotic relationship in corals with high and low rates of bleaching-induced mortality. Coral Reefs.

[16] Sampayo EM, Ridgway T, Bongaerts P, Hoegh-Guldberg O (2008). Bleaching susceptibility and mortality of corals are determined by fine-scale differences in symbiont type. Proc. Natl. Acad. Sci..

[17] Grottoli AG, Rodrigues LJ, Palardy JE (2006). Heterotrophic plasticity and resilience in bleached corals. Nature.

[18] Baird AH, Bhagooli R, Ralph PJ, Takahashi S (2009). Coral bleaching: the role of the host. Trends Ecol. Evol..

[19] Berkelmans R, van Oppen MJ (2006). The role of zooxanthellae in the thermal tolerance of corals: a ‘nugget of hope’ for coral reefs in an era of climate change. Proc. Royal Soc. London B: Biol. Sci..

[20] Putnam HM, Barott KL, Ainsworth TD, Gates RD (2017). The vulnerability and resilience of reef-building corals. Current Biol..

[21] Anthony K, Hoogenboom MO, Maynard JA, Grottoli AG, Middlebrook R (2009). Energetics approach to predicting mortality risk from environmental stress: a case study of coral bleaching. Functional Ecol..

[22] Jones A, Berkelmans R (2010). Potential costs of acclimatization to a warmer climate: growth of a reef coral with heat tolerant vs. sensitive symbiont types. PloS One.

[23] Palumbi SR, Barshis DJ, Traylor-Knowles N, Bay RA (2014). Mechanisms of reef coral resistance to future climate change. Science.

[24] Grottoli AG (2014). The cumulative impact of annual coral bleaching can turn some coral species winners into losers. Global Change Biol..

[25] Loya Y (2001). Coral bleaching: the winners and the losers. Ecol. Lett..

[26] van Woesik R, Sakai K, Ganase A, Loya Y (2011). Revisiting the winners and the losers a decade after coral bleaching. Mar. Ecol. Prog. Ser..

[27] Dudgeon SR, Aronson RB, Bruno JF, Precht WF (2010). Phase shifts and stable states on coral reefs. Mar. Ecol. Prog. Ser..

[28] Fabricius, K. & Alderslade, P. *Soft Corals and Sea Fans: A Comprehensive Guide to the Tropical Shallow Water Genera of the Central-West Pacific*, *the Indian Ocean and the Red Sea*. (AIMS, 2001).

[29] Ribes M, Coma R, Gili JM (1998). Heterotrophic feeding by gorgonian corals with symbiotic zooxanthella. Limnol. Oceanogr..

[30] Fabricius KE, Klumpp DW (1995). Widespread mixotrophy in reef-inhabiting soft corals: the influence of depth, and colony expansion and contraction on photosynthesis. Mar. Ecol. Prog. Ser..

[31] Goulet TL, Simmons C, Goulet D (2008). Worldwide biogeography of *Symbiodinium* in tropical octocorals. Mar. Ecol. Prog. Ser..

[32] Baker DM (2015). Productivity links morphology, symbiont specificity and bleaching in the evolution of Caribbean octocoral symbioses. ISME J..

[33] Poland DÁ, Coffroth MA (2017). Trans-generational specificity within a cnidarian–algal symbiosis. Coral Reefs.

[34] Goulet TL, Coffroth MA (2003). Stability of an octocoral-algal symbiosis over time and space. Mar. Ecol. Prog. Ser..

[35] Michalek-Wagner K, Willis BL (2001). Impacts of bleaching on the soft coral *Lobophytum compactum*. II. Biochemical changes in adults and their eggs. Coral Reefs.

[36] Michalek-Wagner K, Willis BL (2001). Impacts of bleaching on the soft coral *Lobophytum compactum*. I. Fecundity, fertilization and offspring viability. Coral Reefs.

[37] Slattery M (2008). Hybrid vigor in a tropical Pacific soft coral community. Ecol. Monogr..

[38] Slattery M, Gochfeld DJ (2016). Butterflyfishes exhibit species-specific responses to changes in Pacific coral reef benthic communities. Mar. Biol..

[39] Slattery M, Renegar DA, Gochfeld DJ (2013). Direct and indirect effects of a new disease of alcyonacean soft corals. Coral Reefs.

[40] Slattery M, Paul VJ (2008). Indirect effects of bleaching on predator deterrence in the tropical Pacific soft coral *Sinularia maxima*. Mar. Ecol. Prog. Ser..

[41] Paulay G, Benayahu Y (1999). Patterns and consequences of coral bleaching in Micronesia (Majuro and Guam) in 1992-1994. Micronesica.

[42] Reynolds T, Burdick D, Houk P, Raymundo L, Johnson S (2014). Unprecedented coral bleaching across the Marianas Archipelago. Coral Reefs.

[43] Edmunds PJ (2014). Long-term changes in *Symbiodinium* communities in *Orbicella annularis* in St. John, US Virgin Islands. Mar. Ecol. Prog. Ser..

[44] Cunning R, Yost DM, Guarinello ML, Putnam HM, Gates RD (2015). Variability of *Symbiodinium* communities in waters, sediments, and corals of thermally distinct reef pools in American Samoa. PloS One.

[45] Donner SD, Skirving WJ, Little CM, Oppenheimer M, Hoegh‐Guldberg O (2005). Global assessment of coral bleaching and required rates of adaptation under climate change. Global Change Biol..

[46] Strychar KB, Coates M, Sammarco PW, Piva TJ, Scott PT (2005). Loss of *Symbiodinium* from bleached soft corals *Sarcophyton ehrenbergi*, *Sinularia* sp. and *Xenia* sp. J. Exp. Mar. Biol. Ecol..

[47] Kleypas, J. A., Danabasoglu, G. & Lough, J. M. Potential role of the ocean thermostat in determining regional differences in coral reef bleaching events. *Geophysical Res*. *Lett*. **35**, 10.1029/2007GL032257 (2008).

[48] Lesser MP, Stochaj WR, Tapley DW, Schick JM (1990). Bleaching in coral reef anthozoans: effects of irradiance, ultraviolet radiation, and temperature on the activities of protective enzymes against active oxygen. Coral Reefs.

[49] Rodrigues LJ, Grottoli AG, Lesser MP (2008). Long-term changes in the chlorophyll fluorescence of bleached and recovering corals from Hawaii. J. Exp. Biol..

[50] Rodrigues LJ, Grottoli AG (2007). Energy reserves and metabolism as indicators of coral recovery from bleaching. Limnol. Oceanogr..

[51] Muscatine L, McCloskey LR, Marian RE (1981). Estimating the daily contribution of carbon from zooxanthellae to coral animal respiration. Limnol. Oceanogr..

[52] Fitt WK (1982). Photosynthesis, respiration, and contribution to community productivity of the symbiotic sea anemone *Anthopleura elegantissima* (Brandt, 1835). J. Exp. Mar. Biol. Ecol..

[53] Porter JW, Fitt WK, Spero HJ, Rogers CJ, White MW (1989). Bleaching in reef corals: physiological and stable isotopic responses. Proc. Natl. Acad. Sci..

[54] Baker AC (2003). Flexibility and specificity in coral-algal symbiosis: diversity, ecology, and biogeography of *Symbiodinium*. Ann. Rev. Ecol. Evol. Syst..

[55] Lesser MP, Stat M, Gates RD (2013). The endosymbiotic dinoflagellates (*Symbiodinium* sp.) of corals are parasites and mutualists. Coral Reefs.

[56] van de Water, J. A., Allemand. D. & Ferrier-Pagès, C. Host-microbe interactions in octocoral holobionts-recent advances and perspectives. *Microbiome* **6**, 10.1186/s40168-018-0431-6 (2018) 10.1186/s40168-018-0431-6 PMC5880021 29609655

[57] Suggett DJ, Warner ME, Leggat W (2017). Symbiotic dinoflagellate functional diversity mediates coral survival under ecological crisis. Trends Ecol. Evol..

[58] Ulstrup KE, Berkelmans R, Ralph PJ, van Oppen M (2006). Variation in bleaching sensitivity of two coral species across a latitudinal gradient on the Great Barrier Reef: the role of zooxanthellae. Mar. Ecol. Prog. Ser..

[59] Goulet TL, LaJeunesse TC, Fabricius KE (2008). Symbiont specificity and bleaching susceptibility among soft corals in the 1998 Great Barrier Reef mass coral bleaching event. Mar. Biol..

[60] Rowan R (2004). Coral bleaching: thermal adaptation in reef coral symbionts. Nature.

[61] van Oppen MJH, Mieog JC, Sanchez CA, Fabricius KE (2005). Diversity of algal endosymbionts (zooxanthellae) in octocorals: the roles of geography and host relationships. Molecular Ecol..

[62] Prada C (2014). Cryptic diversity hides host and habitat specialization in a gorgonian‐algal symbiosis. Molecular Ecol..

[63] Rowan R, Knowlton N, Baker A, Jara J (1997). Landscape ecology of algal symbionts creates variation in episodes of coral bleaching. Nature.

[64] Davy SK, Allemand D, Weis VM (2012). Cell biology of cnidarian-dinoflagellate symbiosis. Microbiol. Mol. Biol. Rev..

[65] Liu. G, Strong AE, Skirving W, Arzayus LF (2006). Overview of NOAA coral reef watch program’s near-real time satellite global coral bleaching monitoring activities. Proc. 10th Intl. Coral Reef Symp..

[66] NOAA Coral Reef Watch (2013, updated daily) NOAA coral reef watch daily global 5-km satellite virtual station time series data for Guam, 12 Mar 2013 – 31 Dec 2017. College Park MD, USA. Data set accessed: 15 Mar 2018 at, http://coralreefwatch.noaa.gov/vs/index.php.

[67] Eakin CM (2010). Caribbean corals in crisis: record thermal stress, bleaching, and mortality in 2005. PloS One.

[68] Piniak GA, Brown EK (2008). Growth and mortality of coral transplants (*Pocillopora damicornis*) along a range of sediment influence in Maui, Hawaii. Pacific Sci..

[69] Warner, M. E., Lesser, M. P. & Ralph, P. J. Chlorophyll fluorescence in reef building corals. In *Chlorophyll a Fluorescence in Aquatic Sciences: Methods and Applications* 209–222 (Springer, 2010).

[70] Pochon X, Pawlowski J, Zaninetti L, Rowan R (2001). High genetic diversity and relative specificity among *Symbiodinium*-like endosymbiotic dinoflagellates in soritid foraminiferans. Mar. Biol..

[71] Stat M, Loh WKW, LaJeunesse TC, Hoegh-Guldberg O, Carter DA (2009). Stability of coral–endosymbiont associations during and after a thermal stress event in the southern Great Barrier Reef. Coral Reefs.

[72] Comeau AM, Douglas GM, Langille MGI (2017). Microbiome helper: a custom and streamlined workflow for microbiome research. MSystems.

[73] Fu L, Niu B, Wu S, Li W (2012). CD-HIT: accelerated for clustering the next-generation sequencing data. Bioinformatics.

[74] Arif C (2014). Assessing *Symbiodinium* diversity in scleractinian corals via next‐generation sequencing‐based genotyping of the ITS2 rDNA region. Molecular Ecol..

[75] Katoh K, Misawa K, Kuma K, Miyata T (2002). MAFFT: a novel method for rapid multiple sequence alignment based on fast Fourier transform. Nucleic Acids Res..

[76] Stamatakis A (2014). RAxML version 8: a tool for phylogenetic analysis and post-analysis of large phylogenies. Bioinformatics.

[77] Oksanen, J., Blanchet, F. G., Kindt, R., Oksanen, M. J. & Suggests, M. A. Package ‘vegan’. *Community Ecology Package*, *version* **2**(**9**) (2013).

[78] Ripley, B. *et al*. Package ‘mass’. *Cran Repos*. *Httpcran R-Proj* (2013).

