# Supplementary material for: Annual Thermal Stress Increases a Soft Coral’s Susceptibility to Bleaching
Source: Sci Rep. 2019 May 30;9:8064. doi: 10.1038/s41598-019-44566-9 (PMC6542812; doi:10.1038/s41598-019-44566-9)
Supplement: Supplementary file 1 — Supplementary Info [file 41598_2019_44566_MOESM1_ESM.docx]

SUPPLEMENTAL MATERIAL FOR:

Annual Thermal Stress Increases a Soft Coral’s Susceptibility to Bleaching

Marc Slattery^1^, M. Sabrina Pankey^2^, and Michael P. Lesser^2,3^

^1^University of Mississippi, Department of BioMolecular Sciences, Oxford, MS, 38677, USA

^2^University of New Hampshire, Molecular, Cellular, and Biomedical Sciences, Durham, NH, 03824, USA

^3^University of New Hampshire, School of Marine Science and Ocean Engineering, Durham, NH, 03824, USA

S Figure 1. Degree Heating Weeks for Guam. Reprinted data from NOAA Coral Reef Watch shows SSTs and DHWs from 2013 - 2017. These data support the similar intensities of bleaching events in 2013, 2014, and 2017. The dates above each figure indicate reported soft coral bleaching dates at PBH from citizen scientists affiliated with Eyes of the Reef Marianas ([www.eormarianas.org](http://www.eormarianas.org/)). Note: these only indicate the date of observation (i.e., bleaching may pre-date a reported observation), and do not specify which soft coral species were impacted. However, posted photos do demonstrate that *Sinularia* spp. were bleached by the indicated dates.

S Figure 2. Monthly average irradiance for PBH Guam. Downwelling irradiance (E_d_) of photosynthetically active radiation (PAR: 400-700 nm) were collected monthly on replicate (n=3) HOBO Pendant temperature/light loggers at a depth of ~1 m. Presented are the mean ± 1SD irradiance (µmol quanta m^-2^ s^-1^) for May 2016 to December 2017. Data were converted from lumens m^-2^  as described in Piniak & Brown (2008) Pacific Sci 62:39-55. Arrows/dates indicate the approximate onset of soft coral bleaching event (see Fig. S1).

S Figure 3. Average windspeed for PBH Guam. Plotted are the mean daily windspeeds (m^-s^) from March 2013 to December 2017 using NOAA Coral Reef Watch products. The dashed line equates to the doldrums threshold value (<3 m^-s^). Included in this dataset are four periods where doldrum conditions existed for ≥3 successive days: 29 September – 4 October 2016, 21 – 26 August 2017, 28 – 30 September 2017, and 3 – 5 October 2017. Arrows indicate the approximate dates of bleaching events (see Fig. S1).

S Figure 4. Relative abundances of MiSeq reads assigned to most common Symbiodiniaceae variants, across all soft coral samples. Samples are grouped by *Sinularia* species and then by collection year (white: 2016; gray: 2017). Each color reflects a unique *Cladocopium* variant (“CL_xxx’) resulting from CD-HIT clustering at minimum similarity 99%. The *Cladocopium* lineage into which each variant was phylogenetically placed is indicated in parentheses (see tree in S Figure 2). It is also possible that one, or more, of the C3 phylotypes recovered here is *Cladocopium* *thermophilum*. Only variants recovering, on average, more than 20 reads per sample are shown here. The entire list of variants is provided in S Table 3.

S Figure 5. Maximum likelihood phylogeny of the *Cladocopium* lineage (Formerly *Symbiodinium* ITS2 Clade C sequence variants). Datasets for the reference *Cladocopium* lineage and for sequence variants recovered from *Sinularia* have each been clustered to 99% similarity. Bootstrap support is indicated by circle size. Black: Reference sequences; green: *Sinularia* variants significantly enriched in *S. maxima*/hybrid; red: *Sinularia* variants significantly enriched in *S. polydactlya;* blue: *Sinularia* variants not differing in abundance across species.

S Table 1. ANOVA post hoc summary tables. Data presented are the Least Squares Fit Model for the percent of each soft coral population bleached in each year (upper table; Fig. 3a), and the percent cover of each soft coral populations in each year (lower table; Fig 3b). Species groups with same letter are not significantly different by Tukey’s HSD post hoc tests.

| YEAR | SPECIES | GROUP | LEAST SQUARE MEANS |
| --- | --- | --- | --- |
| 2013 | *S. maxima* | F | -6.39e-14 |
| 2013 | *S. polydactyla* | F | 2.84e-14 |
| 2013 | *S. maxima x polydactyla* | F | 5.59e-14 |
| 2014 | *S. maxima* | C | 50.13 |
| 2014 | *S. polydactyla* | F | 3.00 |
| 2014 | *S. maxima x polydactyla* | D | 32.50 |
| 2015 | *S. maxima* | B | 73.38 |
| 2015 | *S. polydactyla* | F | 5.00 |
| 2015 | *S. maxima x polydactyla* | C | 49.25 |
| 2016 | *S. maxima* | A | 90.75 |
| 2016 | *S. polydactyla* | E | 20.50 |
| 2016 | *S. maxima x polydactyla* | B | 75.25 |
| 2017 | *S. maxima* | A | 97.25 |
| 2017 | *S. polydactyla* | CD | 42.25 |
| 2017 | *S. maxima x polydactyla* | A | 93.38 |

| YEAR | SPECIES | GROUP | LEAST SQUARE MEANS |
| --- | --- | --- | --- |
| 2013 | *S. maxima* | C | 20.25 |
| 2013 | *S. polydactyla* | B | 48.13 |
| 2013 | *S. maxima x polydactyla* | D | 5.86 |
| 2014 | *S. maxima* | D | 2.06 |
| 2014 | *S. polydactyla* | AB | 56.75 |
| 2014 | *S. maxima x polydactyla* | D | 1.46 |
| 2015 | *S. maxima* | D | 1.48 |
| 2015 | *S. polydactyla* | A | 57.75 |
| 2015 | *S. maxima x polydactyla* | D | 1.64 |
| 2016 | *S. maxima* | D | 0.13 |
| 2016 | *S. polydactyla* | AB | 51.25 |
| 2016 | *S. maxima x polydactyla* | D | 0.09 |
| 2017 | *S. maxima* | D | 0.09 |
| 2017 | *S. polydactyla* | C | 22.88 |
| 2017 | *S. maxima x polydactyla* | D | 0.08 |

S Table 2. Soft coral percent cover and bleached. Data presented are the total number of soft coral colonies counted on 10 x 2 m transects (n=5 replicates per grid) at PBH Guam, and the total number of bleached colonies within the population.

| YEAR | SPECIES | COLONIES | BLEACHED COLONIES |
| --- | --- | --- | --- |
| 2013 | *S. maxima* | 186 | 0 |
| 2013 | *S. polydactyla* | 473 | 0 |
| 2013 | *S. maxima x polydactyla* | 49 | 0 |
| 2014 | *S. maxima* | 25 | 12 |
| 2014 | *S. polydactyla* | 501 | 13 |
| 2014 | *S. maxima x polydactyla* | 22 | 7 |
| 2015 | *S. maxima* | 24 | 19 |
| 2015 | *S. polydactyla* | 492 | 25 |
| 2015 | *S. maxima x polydactyla* | 22 | 11 |
| 2016 | *S. maxima* | 19 | 17 |
| 2016 | *S. polydactyla* | 461 | 35 |
| 2016 | *S. maxima x polydactyla* | 19 | 15 |
| 2017 | *S. maxima* | 17 | 17 |
| 2017 | *S. polydactyla* | 238 | 107 |
| 2017 | *S. maxima x polydactyla* | 16 | 15 |

S Table 3. ANOVA post hoc summary tables. Data presented are the Least Squares Fit Model for the percent area of each soft coral colony bleached in each year (upper table; Fig. 4a), and the average quantum yield (Fv/Fm) of each soft coral colony in each year (lower table; Fig. 4b). Species groups with same letter are not significantly different by Tukey’s HSD post hoc tests.

| YEAR | SPECIES | GROUP | LEAST SQUARE MEANS |
| --- | --- | --- | --- |
| 2014 | *S. maxima* | A | 79.88 |
| 2014 | *S. polydactyla* | E | 1.13 |
| 2014 | *S. maxima x polydactyla* | AB | 70.50 |
| 2015 | *S. maxima* | DE | 10.38 |
| 2015 | *S. polydactyla* | E | 2.00 |
| 2015 | *S. maxima x polydactyla* | E | 4.88 |
| 2016 | *S. maxima* | D | 20.00 |
| 2016 | *S. polydactyla* | DE | 10.00 |
| 2016 | *S. maxima x polydactyla* | DE | 10.13 |
| 2017 | *S. maxima* | A | 75.00 |
| 2017 | *S. polydactyla* | C | 49.75 |
| 2017 | *S. maxima x polydactyla* | BC | 60.00 |

| YEAR | SPECIES | GROUP | LEAST SQUARE MEANS |
| --- | --- | --- | --- |
| 2013 | *S. maxima* | A | 0.74 |
| 2013 | *S. polydactyla* | A | 0.76 |
| 2013 | *S. maxima x polydactyla* | A | 0.74 |
| 2014 | *S. maxima* | C | 0.54 |
| 2014 | *S. polydactyla* | A | 0.76 |
| 2014 | *S. maxima x polydactyla* | CD | 0.51 |
| 2015 | *S. maxima* | EF | 0.47 |
| 2015 | *S. polydactyla* | A | 0.76 |
| 2015 | *S. maxima x polydactyla* | DE | 0.48 |
| 2017 | *S. maxima* | G | 0.39 |
| 2017 | *S. polydactyla* | B | 0.59 |
| 2017 | *S. maxima x polydactyla* | FG | 0.42 |

S Table 4. ANOVA results for effect of species and collection year on abundance of each unique *Symbiodinium* variant recovered from ITS2 amplicon sequencing. P-values are Bonferroni corrected; significant variants are indicated in bold. Variants are listing in descending order based on their mean proportional abundance across all samples. Of all 71 unique variants recovered, 13 differ significantly across species (~18%).

| ***Symbiodinium*** **variant** | **Mean relative abundance** | **SPECIES** | | **YEAR** | |
| --- | --- | --- | --- | --- | --- |
|  |  | **F** | **p-value** | **F** | **p-value** |
| **CL_172** | **0.4495** | **24.6896** | **0** | **0.0504** | **1** |
| **CL_174** | **0.185** | **10.6443** | **0.021** | **3.377** | **1** |
| CL_170 | 0.1547 | 8.0437 | 0.095 | 5.7004 | 1 |
| CL_178 | 0.0662 | 0.6385 | 1 | 2.7863 | 1 |
| CL_180 | 0.0406 | 2.9956 | 1 | 1.0313 | 1 |
| CL_175 | 0.0278 | 1.8827 | 1 | 1.7043 | 1 |
| CL_265 | 0.0116 | 6.4415 | 0.254 | 2.1807 | 1 |
| CL_193 | 0.0191 | 1.3359 | 1 | 0.4545 | 1 |
| CL_89 | 0.0087 | 1.4201 | 1 | 1.9262 | 1 |
| CL_185 | 0.0062 | 1.166 | 1 | 0.881 | 1 |
| **CL_210** | **0.0052** | **13.8897** | **0.004** | **1.0019** | **1** |
| CL_1 | 0.0024 | 5.8992 | 0.347 | 7.8658 | 0.623 |
| CL_85 | 0.003 | 48.8832 | 0 | 0.0318 | 1 |
| CL_192 | 0.0035 | 0.8716 | 1 | 1.0188 | 1 |
| **CL_274** | **0.0013** | **9.8463** | **0.033** | **0.1728** | **1** |
| CL_247 | 0.0024 | 2.6486 | 1 | 0.0817 | 1 |
| **CL_317** | **0.0012** | **14.9476** | **0.002** | **0.9366** | **1** |
| CL_217 | 0.0016 | 3.7547 | 1 | 0.1502 | 1 |
| CL_361 | 0.0008 | 3.8096 | 1 | 0.0284 | 1 |
| CL_186 | 0.0006 | 1.0647 | 1 | 1.0625 | 1 |
| **CL_280** | **0.0005** | **13.5017** | **0.005** | **1.9133** | **1** |
| CL_261 | 0.0007 | 5.5774 | 0.419 | 0.7106 | 1 |
| **CL_208** | **0.0006** | **16.5833** | **0.001** | **4.2403** | **1** |
| CL_277 | 0.0005 | 2.0114 | 1 | 1.0426 | 1 |
| CL_257 | 0.0006 | 1.1925 | 1 | 0.9976 | 1 |
| CL_236 | 0.0005 | 5.3535 | 0.483 | 0.1526 | 1 |
| CL_305 | 0.0004 | 3.5797 | 1 | 1.1709 | 1 |
| CL_249 | 0.0004 | 0.9043 | 1 | 1.0921 | 1 |
| CL_3 | 0.0003 | 3.8692 | 1 | 1.1438 | 1 |
| **CL_230** | **0.0003** | **10.326** | **0.025** | **3.2277** | **1** |
| **CL_238** | **0.0003** | **11.9375** | **0.01** | **0.277** | **1** |
| CL_259 | 0.0003 | 4.5669 | 0.811 | 0.0067 | 1 |
| CL_194 | 0.0003 | 1.7532 | 1 | 0.4204 | 1 |
| CL_367 | 0.0003 | 5.8762 | 0.347 | 1.2323 | 1 |
| CL_206 | 0.0002 | 4.4677 | 0.855 | 0.7849 | 1 |
| CL_211 | 0.0002 | 3.376 | 1 | 0.4644 | 1 |
| CL_254 | 0.0002 | 0.9969 | 1 | 1.1421 | 1 |
| CL_294 | 0.0002 | 3.7803 | 1 | 0.5894 | 1 |
| CL_250 | 0.0002 | 1.9328 | 1 | 1.2145 | 1 |
| CL_214 | 0.0001 | 2.9294 | 1 | 0.4882 | 1 |
| CL_191 | 0.0001 | 3.9313 | 1 | 0.5021 | 1 |
| CL_332 | 0.0001 | 2.5469 | 1 | 1.3373 | 1 |
| CL_198 | 0.0001 | 2.3851 | 1 | 1.4876 | 1 |
| CL_284 | 0.0001 | 3.5606 | 1 | 0.1102 | 1 |
| CL_285 | 0.0001 | 2.0429 | 1 | 1.0872 | 1 |
| CL_312 | 0.0001 | 7.5657 | 0.125 | 0.7448 | 1 |
| CL_298 | 0.0001 | 3.0846 | 1 | 0.1946 | 1 |
| CL_205 | 0.0001 | 4.874 | 0.659 | 2.2746 | 1 |
| CL_309 | 0.0001 | 2.6842 | 1 | 1.509 | 1 |
| CL_439 | 0.0001 | 3.9104 | 1 | 0.5841 | 1 |
| CL_207 | 0.0001 | 3.5474 | 1 | 2.0023 | 1 |
| CL_816 | 0.0001 | 1.1267 | 1 | 0.2583 | 1 |
| CL_291 | 0.0001 | 3.7894 | 1 | 0.5158 | 1 |
| CL_345 | 0.0001 | 1.1707 | 1 | 2.258 | 1 |
| CL_484 | 0.0001 | 6.4593 | 0.254 | 0.0025 | 1 |
| CL_276 | > 0.0001 | 6.5925 | 0.236 | 1.1057 | 1 |
| **CL_333** | **> 0.0001** | **18.0134** | **0.001** | **0.0149** | **1** |
| CL_258 | > 0.0001 | 2.6928 | 1 | 0.7801 | 1 |
| **CL_340** | **> 0.0001** | **9.8141** | **0.033** | **0.8674** | **1** |
| CL_1014 | > 0.0001 | 6.2957 | 0.273 | 1.0034 | 1 |
| CL_252 | > 0.0001 | 7.6035 | 0.124 | 0.6525 | 1 |
| **CL_200** | **> 0.0001** | **8.2744** | **0.083** | **0** | **1** |
| CL_244 | > 0.0001 | 2.6789 | 1 | 0.0396 | 1 |
| CL_5 | > 0.0001 | 6.2596 | 0.275 | 0.5229 | 1 |
| CL_304 | > 0.0001 | 4.4 | 0.879 | 0.3849 | 1 |
| CL_306 | > 0.0001 | 5.0986 | 0.57 | 0.691 | 1 |
| **CL_330** | **> 0.0001** | **12.2079** | **0.009** | **10.5625** | **0.207** |
| CL_533 | > 0.0001 | 2.1285 | 1 | 0.0938 | 1 |
| CL_5348 | > 0.0001 | 2.5203 | 1 | 0.8781 | 1 |
| CL_295 | > 0.0001 | 3.1987 | 1 | 1.0449 | 1 |
| CL_548 | > 0.0001 | 2.8918 | 1 | 0.14 | 1 |

S Table 5. PERMANOVA post-hocs tests for pairwise differences in *Symbiodinium* diversity among *Sinularia maxima* (*Sm*), *Sinularia polydactyla* (*Sp*), and the hybrid of these two soft corals.

|  | Df | F.Model | R2 | Pr(>F) |
| --- | --- | --- | --- | --- |
| *Sm* vs *Sp* | 1 | 15.5237 | 0.37746 | 0.0001 |
| year | 1 | 5.8814 | 0.14301 | 0.0012 |
| species x year | 1 | 2.7216 | 0.06618 | 0.0503 |
| residuals | 17 |  | 0.41336 |  |

|  | Df | F.Model | R2 | Pr(>F) |
| --- | --- | --- | --- | --- |
| hybrid vs *Sp* | 1 | 19.9268 | 0.39922 | 0.0001 |
| year | 1 | 4.7350 | 0.09486 | 0.0035 |
| species x year | 1 | 5.2527 | 0.10523 | 0.0019 |
| residuals | 20 |  | 0.40069 |  |

|  | Df | F.Model | R2 | Pr(>F) |
| --- | --- | --- | --- | --- |
| hybrid vs *Sm* | 1 | 1.02806 | 0.04839 | 0.3588 |
| year | 1 | 0.88765 | 0.04178 | 0.4492 |
| species x year | 1 | 2.32840 | 0.10960 | 0.0495 |
| residuals | 17 |  | 0.80022 |  |

S Table 6. PERMANOVA results for effect of observation year on *Symbiodinium* variation within each *Sinularia* parental species and hybrid.

| *HYBRID* | Df | F.Model | R2 | Pr(>F) |
| --- | --- | --- | --- | --- |
| Year | 1 | 2.6534 | 0.2097 | 0.098 |
| Residuals | 10 |  | 0.7903 |  |

| *S. maxima* | Df | F.Model | R2 | Pr(>F) |
| --- | --- | --- | --- | --- |
| Year | 1 | 1.105 | 0.13633 | 0.3437 |
| Residuals | 7 |  | 0.86367 |  |

| *S. polydactyla* | Df | F.Model | R2 | Pr(>F) |
| --- | --- | --- | --- | --- |
| Year | 1 | 0.56169 | 0.43831 | 0.0073 |
| Residuals | 10 |  | 0.56169 |  |

S Table 7. PERMANOVA results for effects of *Sinularia* species and treatment on *Symbiodinium* variation among natural and manipulated 2017 samples.

|  | Df | F.Model | R2 | Pr(>F) |
| --- | --- | --- | --- | --- |
| species | 2 | 6.9919 | 0.50599 | 0.001 |
| treatment | 1 | 1.0556 | 0.03820 | 0.329 |
| species x treatment | 2 | 0.2987 | 0.02162 | 0.964 |
| Residuals | 12 |  | 0.43420 |  |
